# High IgG titers against EBV glycoprotein 42 correlate with lower risk of nasopharyngeal carcinoma

**DOI:** 10.1172/JCI189207

**Published:** 2025-02-17

**Authors:** Benjamin E. Warner, Kathy H.Y. Shair

**Affiliations:** 1 Cancer Virology Program, UPMC Hillman Cancer Center,; 2 Cancer Epidemiology and Prevention Program, UPMC Hillman Cancer Center, and; 3 Department of Microbiology and Molecular Genetics, University of Pittsburgh, Pittsburgh, Pennsylvania, USA.

## Abstract

Serologic biomarkers for the early diagnosis of EBV-associated nasopharyngeal carcinoma (NPC) have been identified from population studies, but a protective antibody signature in cancer-free seropositive carriers remains undefined. In this issue of the *JCI*, Kong et al. show that high levels of IgG against EBV glycoprotein 42 (gp42) were associated with reduced NPC risk in three independent prospective cohorts from southern China. EBV virions contain gp42, which complexes with gH-gL to facilitate fusion with B cells by binding to HLA class II (HLA-II). In this study, HLA-II was detected on non-antigen-presenting cells in a proportion of premalignant nasopharyngeal tissues, which may prime the nasopharyngeal epithelium for infection. In vitro, HLA-II expression in a nasopharyngeal cell line encouraged infection by EBV derived from B cells or epithelial cells. These findings suggest that a vaccine that stimulates gp42-IgG production may reduce the risk of EBV-associated NPC in endemic regions.

## Antibody-based screening for nasopharyngeal carcinoma

**Figure 1:**
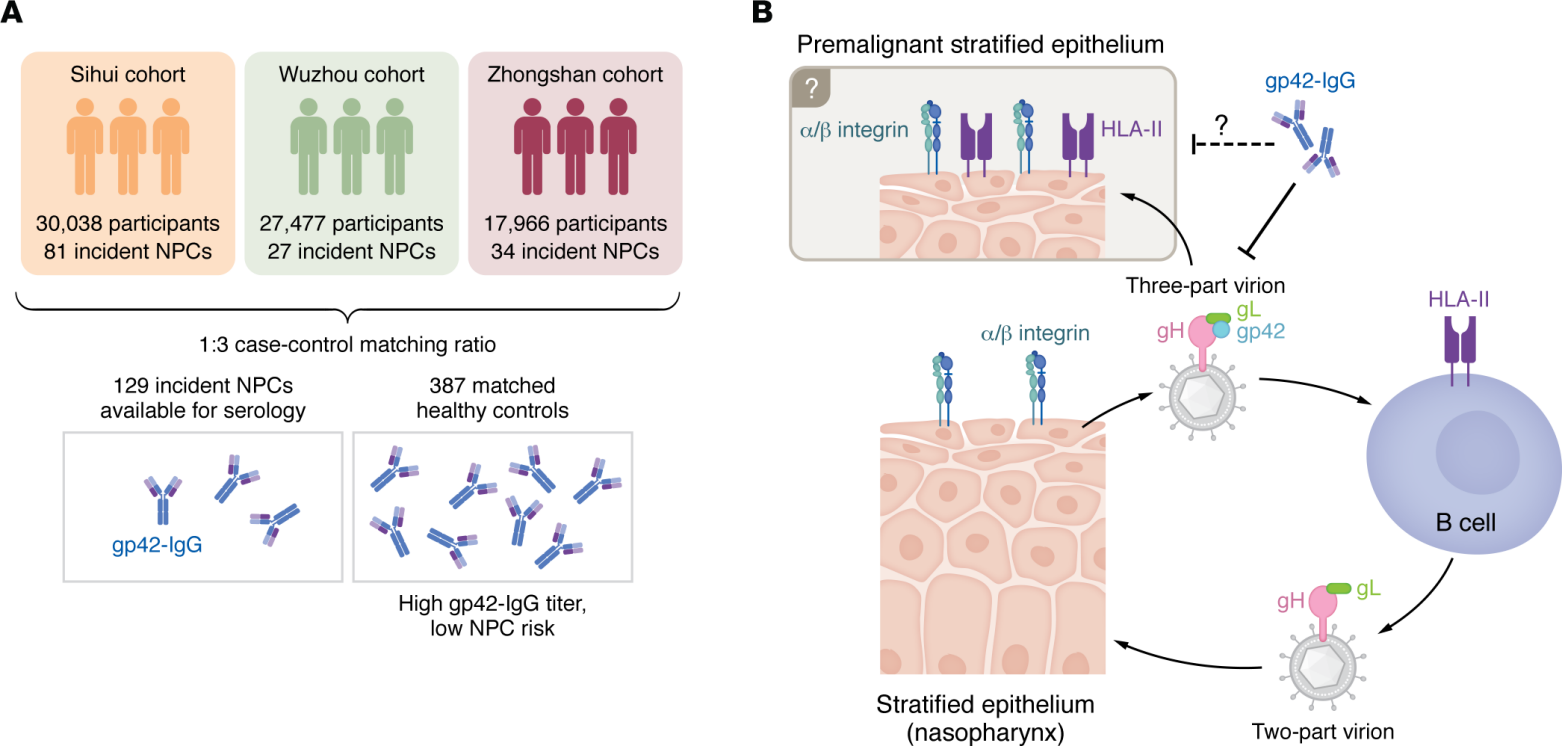
Elevated levels of gp42-IgG are associated with a reduced risk of NPC. (**A**) Serologic screen of incident NPC and matched healthy controls (1:3 ratio) from three independent prospective cohorts demonstrated individuals with higher titers of IgG against EBV gp42 (gp42-IgG) had a lower risk of NPC diagnosis. (**B**) Alternate replication in stratified epithelial cells and B cells produce virions that selectively target the reciprocal cell type. Kong and co-authors proposed that HLA-II primes the premalignant tissue for infection by three-part virions produced in the oral and nasal epithelium (9). In this model, abundant gp42-IgG could reduce the risk of EBV infection by interfering with membrane fusion.

EBV infection is closely associated with nasopharyngeal carcinoma (NPC) in endemic regions such as southern China (1). Screening populations for high-risk antibody signatures can reduce the burden of NPC by improving early diagnosis (2, 3). For example, mucosa-specific antibodies (IgA) against EBV proteins, such as EBNA1 and VCA viral capsid antigen (VCA) p18 and systemic antibodies (IgG) against BNLF2b can distinguish healthy individuals from those with early-stage NPC (2, 4, 5). Their utility in risk assessment has been likewise evaluated by profiling antibodies in healthy individuals who were later diagnosed with NPC (incident cases) (6–8). While such studies have revealed biomarkers of NPC risk, a protective antibody signature in healthy EBV carriers in NPC-endemic regions remains unclear. In this issue of the *JCI*, Kong et al. report that high levels of IgG against EBV glycoprotein 42 (gp42) were associated with reduced NPC risk across three independent prospective cohorts in southern China (Figure 1A) (9). Most adults are seropositive for EBV, but whether healthy carriers harbor serologic markers that correlate with a lower odds ratio for NPC is unknown. The authors quantified gp42-IgG by an ELISA coated with mammalian-expressed purified gp42 lacking the transmembrane region. Individuals with low gp42-IgG were at greater risk of NPC up to 5 years before diagnosis compared with healthy controls. This finding is intriguing because it suggests that neutralizing gp42-IgG may reduce the frequency of EBV infections in the nasopharynx and, by extension, the risk of developing NPC. Cytotoxic T lymphocyte (CTL) responses are important for the recognition of EBV antigens in NPC (10), but a role for humoral immunity in protection against cancer is as yet undefined. Emerging evidence from spatial transcriptomics have indicated that antibody-producing plasma cells are associated with antitumor apoptotic signatures (11).

Recent studies investigating neutralizing antibody levels and NPC risk have offered conflicting results (12–16). A previous case-control study by the same group using a smaller but different subset of plasma samples from the Sihui cohort (20 cases, 40 controls) did not show that gp42-IgG or -IgA levels correlated with NPC protection or neutralizing activity in B cell or epithelial cell lines (14). The 129 incident NPC cases and 387 controls across three cohorts in the present study is one of the largest prospective case-control studies to date (9, 17). The authors present in vitro and hisopathologic evidence that supports a proposed mechanism of gp42-IgG protection. Competition with soluble gp42 inhibited EBV infection of epithelial cells expressing HLA class II (HLA-II) (9). Indeed, a preclinical study showed that monoclonal antibodies targeting gp42 can directly inhibit HLA-II binding to neutralize B cell infection (18), but the in vivo relevance of neutralizing gp42 antibody and infection in the nasopharyngeal epithelium is unknown. A strong neutralizing antibody response can be detected during primary EBV infection and infectious mononucleosis (19), but cell-mediated immunity is thought to have a greater role in controlling disease associated with EBV reactivation (20, 21). Thus, exploring systems serology in the control of EBV infection prior to NPC development may confirm or reveal new targets for vaccine design. Current vaccine efforts are targeted at stimulating neutralizing antibodies against EBV surface antigens that will most likely reduce infection burden but may not be sterilizing. Whether these vaccines protect against cancer resulting from the outgrowth of a latent infection will necessitate the development of cancer models or observational studies from clinical trials (21).

## EBV latency in the nasopharynx

The origin of latent EBV infection in the nasopharynx remains elusive, which could be attributed to the sparse sampling of EBV-infected mucosal epithelial cells (22). EBV is transmitted through saliva and infects the oropharyngeal mucosa and surrounding lymphoid tissue, where it establishes lifelong latency in B cells (23). One hypothesis proposes that EBV infects the nasopharynx during periodic reactivation from infiltrating B cells. Given the alternate tropism of EBV, cell-type–specific production of two- or three-part glycoprotein complexes on the virion will direct the trafficking of EBV between B cells and epithelial cells (Figure 1B) (24). Virions produced in B cells contain predominantly two-part gH-gL complexes, which bind integrins to activate epithelial cell fusion (25). Virions from epithelial cells contain mostly three-part gH-gL-gp42 complexes, which bind HLA-II to promote B cell fusion (26). Abundant HLA-II in B cells restricts gp42 incorporation into progeny virions (24). Consequently, virions produced in B cells show a greater propensity to infect epithelial cells and vice versa. Kong et al. provide evidence that the presumed HLA-II–negative nasopharyngeal tissue can in fact express HLA-II in individuals presenting with atypical dysplasia (9). One can surmise that an inflammatory environment could lead to the aberrant expression of HLA-II in a non-antigen-presenting cell, as occurs in the inflamed intestinal epithelium (27). Moreover, HLA-II has been previously detected in NPC tissue and was associated with a poor prognosis (28). Thus, the findings of Kong et al. lead to the hypothesis that premalignant nasopharyngeal cells presenting HLA-II are poised for infection by epithelial cell–derived EBV (9). This provides a potential mechanism for gp42-IgG protection, without EBV necessarily shuttling through infiltrating B cells, and might explain the reduction in NPC risk for individuals with elevated gp42-IgG levels (9). In the absence of an NPC premalignant animal model, these conclusions are largely limited to observational studies. The possibility of an association between EBV-neutralizing activity and high gp42-IgG levels was not assessed in Kong et al. and remains a limitation of the study (9). Nevertheless, antibody-dependent responses and/or neutralization of the infectivity of three-part EBV virions could prove to be a vital step in reducing the likelihood of cellular transformation.

## Concluding remarks and perspectives

While the burden of NPC can be reduced by screening at-risk populations, the development of a therapeutic vaccine to reduce NPC incidence remains highly desirable. Many EBV vaccine candidates have focused on targeting glycoproteins, with the goal of neutralizing infection (29, 30). A prophylactic vaccine may not be sterilizing, but persistently high antibody levels could conceivably minimize infections in the upper respiratory tract. What is perhaps unexpected from this study is the histopathological evidence that HLA-II staining was observed in nearly half (13 of 27, 48%) of the sampled premalignant nasopharyngeal tissue (9). Recognizing that curated nasopharyngeal tissue from an asymptomatic control group is hard to come by, this repository of 27 clinic-based premalignant nasopharyngeal tissue offers unique insight into the preneoplastic, possibly inflammatory environment of incident cases. Although clear gp42-IgG cutoffs could not be established to discriminate incident NPCs from healthy controls, binning the test positives by antibody titer revealed that the highest quartile in the control group displayed the lowest OR for NPC risk. Thus, more effort must be dedicated to finding a proxy for correlates of cancer protection. In the absence of a nasopharyngeal tumor development model, observational studies from large prospective cohorts have become a hallmark of NPC research, and the findings of Kong et al. would support the premise that a vaccine that indirectly targets a glycoprotein indirectly has potential in cancer prevention (9).

## Version 1 02/17/2025

Electronic publication
